# A Comparative Study on Antioxidant System in Fish Hepatopancreas and Intestine Affected by Choline Deficiency: Different Change Patterns of Varied Antioxidant Enzyme Genes and Nrf2 Signaling Factors

**DOI:** 10.1371/journal.pone.0169888

**Published:** 2017-01-18

**Authors:** Pei Wu, Yang Liu, Wei-Dan Jiang, Jun Jiang, Juan Zhao, Yong-An Zhang, Xiao-Qiu Zhou, Lin Feng

**Affiliations:** 1 Animal Nutrition Institute, Sichuan Agricultural University, Chengdu, China; 2 Fish Nutrition and Safety Production University Key Laboratory of Sichuan Province, Sichuan Agricultural University, Chengdu, China; 3 Key Laboratory for Animal Disease-Resistance Nutrition of China Ministry of Education, Sichuan Agricultural University, Chengdu, China; 4 Institute of Hydrobiology, Chinese Academy of Sciences, Wuhan, China; Max Delbrueck Center for Molecular Medicine, GERMANY

## Abstract

The liver and intestine are susceptible to the oxidative damage which could result in several diseases. Choline deficiency induced oxidative damage in rat liver cells. Thus, this study aimed to investigate the potential molecular mechanisms responsible for choline deficiency-induced oxidative damage. Juvenile Jian carp were fed diets differing in choline content [165 (deficient group), 310, 607, 896, 1167 and 1820 mg/kg diet] respectively for 65 days. Oxidative damage, antioxidant enzyme activities and related gene expressions in the hepatopancreas and intestine were measured. Choline deficiency decreased choline and phosphatidylcholine contents, and induced oxidative damage in both organs, as evidenced by increased levels of oxidative-stress markers (malondialdehyde, protein carbonyl and 8-hydroxydeoxyguanosine), coupled with decreased activities of antioxidant enzymes [Copper-zinc superoxide dismutase (CuZnSOD), manganese superoxide dismutase (MnSOD), glutathione peroxidase (GPx) and glutathione-S-transferase (GST)]. However, choline deficiency increased glutathione contents in the hepatopancreas and intestine. Furthermore, dietary choline deficiency downregulated mRNA levels of *MnSOD*, *GPx1b*, *GST-rho*, *mGST3* and *Kelch-like ECH associating protein 1* (*Keap1b*) in the hepatopancreas, *MnSOD*, *GPx1b*, *GPx4a*, *GPx4b*, *GST-rho*, *GST-theta*, *GST-mu*, *GST-alpha*, *GST-pi* and *GST-kappa* in the intestine, as well as intestinal Nrf2 protein levels. In contrast, choline deficiency upregulated the mRNA levels of *GPx4a*, *GPx4b*, *mGST1*, *mGST2*, *GST-theta*, *GST-mu*, *Keap1a* and *PKC* in the hepatopancreas, *mGST3*, *nuclear factor erythoid 2-related factor 2 (Nrf2)* and *Keap1a* in the intestine, as well as hepatopancreatic Nrf2 protein levels. This study provides new evidence that choline deficiency-induced oxidative damage is associated with changes in the transcription of antioxidant enzyme and Nrf2/Keap1 signaling molecules in the hepatopancreas and intestine. Additionally, this study firstly indicated that choline deficiency induced varied change patterns of different *GPx* and *GST* isoforms. Meanwhile, the changes of some *GPx* and *GST* isoforms caused by choline deficiency in the intestine were contrary to those in the hepatopancreas.

## Introduction

Oxidative stress appears to play a major role in the pathogenesis and progression of many liver and intestinal diseases [1,2]. Therefore, it is very important to enhance the antioxidant ability of liver and intestine thus increasing the liver and intestine health. Recently, dietary nutritional supplements were found to be an efficient method for improving organ antioxidant capacity. Choline is an essential vitamin for humans and other animals [3]. Our previous study showed that choline deficiency induced growth retardation and decreased growth and development of hepatopancreas and intestine in juvenile Jian carp (*Cyprinus curpio* var. Jian) [4]. Furthermore, choline deficiency in rats induced the generation of reactive oxygen species (ROS) in hepatocytes [5], and increased lipid peroxidation in the liver [6]. These results showed that choline deficiency might induce oxidant damage in the liver and intestine. But the detailed effect of choline on antioxidant systems in the liver and intestine and the underlying molecular mechanisms remain largely unknown.

Vertebrates possess enzymatic and non-enzymatic antioxidant systems as defense against oxidative stress [7]. Our previous study showed that activities of superoxide dismutase (SOD), catalase (CAT), glutathione peroxidase (GPx), glutathione-S-transferase (GST) and glutathione reductase (GR) in fish spleen were increased by choline deficiency probably as an adaptive response [8]. The spleen is one of the main immune organs in fish. Reactive oxygen species involves in immune cell functions such as cytotoxic and microbicidal activities [9]. However, the liver (or hepatopancreas in some fish species) is the central metabolic organ, whereas the intestine is an important site for nutrient digestion and absorption. Excessive ROS decreased hepatocytes viability in rat [5] and intestinal epithelial cell function in carp [10]. Thus, the antioxidant response to choline in the liver and intestine might be different from that in the spleen, which is valuable for investigation. In rat, enhanced antioxidant enzyme activity was allied with upregulated gene expression [11]. Some antioxidant enzymes (e.g., GPx and GST) are polygenic. In common carp (*Cyprinus carpio*), *GPx1* and *GPx4* have been identified [12], and nine GST genes (*GST-alpha*, *-kappa*, *-theta*, *-mu*, *-pi*, *-rho*, *mGST1*, *mGST2* and *mGST3*) have been cloned [13]. Despite being in the same family, genes encoding antioxidant enzymes tend to exhibit differing tissue distribution and function. In river pufferfish (*Takifugu obscurus*), *GST-theta*, *-mu*, *-MAPEG* and *-zeta* gene expression in the liver were higher than *GST-alpha*, *-kappa* and *-omega* gene expression [14]. Among GPxs, GPx4 is the only one capable of reducing phospholipid hydroperoxides in vertebrate [12]. Furthermore, a recent study from our laboratory showed that antioxidant enzyme genes in the same family also have varied responses to nutrient content: dietary vitamin C deficiency downregulated gene expressions of *GST-R1*, *-P1* and *-P2*, while not affecting gene expressions of *GST-O1* and *-O2* in the spleen of grass carp (*Ctenopharyngodon idella*) [15]. However, the effect of choline on isoforms of antioxidant enzyme genes has not yet been studied in animals. In mammal, Cu/Zn-SOD, CAT, GST and GR genes contain antioxidant response element (ARE) [16], and the nuclear factor erythoid 2-related factor 2 (Nrf2) can regulate expression of ARE-containing genes [17]. In its basal state, Nrf2 is retained in the cytoplasm by its inhibitor, Kelch-like ECH associating protein 1 (Keap1) [18], but phosphorylation by protein kinase C (PKC) triggers its translocation to the nucleus in mammal [19]. Recently, our laboratory found that Nrf2, Keap1 and PKC are also widely distribute in fish hepatopancreas and intestine, and can be regulated by inositol [20]. To date, whether choline could affect the Nrf2 signaling in liver and intestine is unclear. In terrestrial animal, diacylglycerol (DAG) can activate PKC [21]. Choline deficiency increased DAG content in rat [22]. Therefore, choline might affect Nrf2 signaling in the liver and intestine.

The liver and intestine are involved in different functions and the challenges that they are closely confronted are also different. As the major detoxification organ in vertebrates, the liver is also central to the degradation of metabolic products [23], and thus is constantly challenged by many endogenous and exogenous free radicals. As the main site for nutrients digestion and absorption, the intestine is constantly challenged by diet-derived oxidants, as well as endogenously generated ROS [24]. Furthermore, it is reported that antioxidant defenses are more highly developed in liver than in other organs [25]. In river pufferfish, the expressions of *GST-theta*, *-mu*, *-MAPEG* and *-zeta* genes in the liver were five- to ten-fold higher than those in other tissues [14]. Accordingly, liver and intestine susceptibility to oxidative stress may differ. In vertebrates, the liver and intestine play distinct roles in choline metabolism. The liver is probably the most active organ for choline metabolism [26], while the intestine is the main site for choline absorption [27]. In rat, the liver is the first organ that affected by choline deficiency [28]. These might result in the different susceptibility of liver and intestine to choline deficiency. Hence, it is of interest to look at the antioxidant response of liver and intestine to choline deficiency.

As Cyprinidae fish, the common carp (*Cyprinus carpio*) is closely related to zebrafish (*Danio rerio*), a commonly used animal model to study human disease [29], and is also highly suitable for comparative physiological and disease studies in combination with zebrafish [30]. Jian carp is a strain of carp developed by crossbreeding, inbreeding and artificial selection of common carp, and has genetic similarity with common carp [31]. Thus, we investigated the effect of choline on antioxidant ability of hepatopancreas and intestine in Jian carp. The antioxidant parameters, gene expression of antioxidant enzymes and Nrf2-Keap1 signaling molecules in hepatopancreas and intestine were assessed, aiming to provide partial theoretical evidence for mechanism underlying the effect of choline on antioxidant system, and provide a clue for understanding mechanisms underlying the effect of choline on fatty liver disease and intestinal inflammation.

## Materials and Methods

### Diet manipulation and feeding trial

The present study used one animal trial as our previous study [4]. Formulation and proximate composition of the basal diet is presented in S1 Table. The experimental diet, and the procedures for diet preparation and storage (-20°C) were the same as our previous study [4]. Total sulfur amino acids (TSAA, methionine + cysteine) were formulated to marginally satisfy the TSAA requirement of juvenile Jian carp [32]. Choline chloride (Sigma Chemicals) was added to the basal diet to provide graded levels of choline. The choline concentrations in experimental diets were determined by the method of Venugopal [33], and final choline concentrations of six experimental diets were 165, 310, 607, 896, 1167 and 1820 mg/kg diet.

The feeding trail followed the Guidelines for the Care and Use of Laboratory Animals of Animal Nutritional Institute, Sichuan Agricultural University, and the same as our previous study [4]. Juvenile Jian carp were obtained from Tong Wei Hatchery (Sichuan, China). After one month acclimation, a total of 1200 fish (average initial weight 7.94 ± 0.01g) were randomly distributed into 24 aquaria (90 *L*×30 *W*×40 *H* cm). The aquaria system, water quality, and operation of the culture system were as previously described [4]. For the feeding trial, the six experimental diets were randomly assigned to four aquaria each. Fish were fed to apparent satiation six times daily from 1 to 30 days and four times daily from 31 to 65 days, and uneaten feed was siphoned out after each meal.

### Sampling

At the termination of the feeding trial, fish collected from each aquarium were anaesthetized in benzocaine bath (50 mg/L) after being fasted for 12h as our previous study [4]. The hepatopancreas and intestine of 15 fish were removed, weighed and frozen in liquid nitrogen, then stored at -70°C until analyzed. All procedures were approved by the Institutional Animal Care and Use Committee of Sichuan Agricultural University.

### Measurement of biochemical and antioxidant related parameters

Tissue homogenates of hepatopancreas and intestine were prepared in 10 volumes (w/v) of ice-cold normal saline and centrifuged at 6000 *g* and 4°C for 20 min. The supernatant was conserved and used to determine biochemical and antioxidant parameters. Choline and phosphatidylcholine (PtdCho) contents were measured by the method of enzyme hydrolysis based on Hojjati and Jiang [34]. Protein concentration was determined by the method of Brandford [35]. Malondialdehyde (MDA) and protein carbonyl (PC) contents were measured as the method described by Zhang et al. [36] and Tokur & Korkmaz [37] respectively. 8-hydroxydeoxyguanosine (8-OHdG) content was measured by using a competitive enzyme-linked immunosorbent assay (ELISA) kit (Elabscience) according to the manufacturer’s instructions. ROS content was measured following the method described by Tirosh et al. [38] with slight modification. CuZnSOD and MnSOD activities were determined according to the method of Lambertucci et al. [39]. CAT activity was determined according to Aebi [40]. GPx activity was assayed according to the methods described by Zhang et al. [36]. Activities of GST and GR were measured as described by Lushchak et al. [41] and Lora et al. [42] respectively. Glutathione (GSH) content was determined by the formation of 5-thio-2-nitrobenzoate according to Vardi et al. [43].

### RNA extraction and RT-qPCR

Total RNA was extracted from the hepatopancreas and intestine by using the RNAiso plus Kit (Takara). The purity of RNA was assessed by spectrophotometry at 260 and 280 nm, and electrophoresis on 1% agarose gels. The PrimeScript^™^ RT reagent Kit (Takara) was used to synthesize the first-strand cDNA using 2 μl of total RNA. The quantification of *CuZnSOD*, *MnSOD*, *CAT*, *GPxs*, *GSTs*, *GR*, *Nrf2*, *Keap1a*, *Keap1b*, *PKC* and house-keeping gene (*β-actin*) transcript levels was performed via the real-time quantitative PCR (qPCR) using the CFX96^™^ Real-Time PCR Detection System (Bio-Rad) with SYBR Green (Takara). The reactions followed standard protocols with primers and thermocycling conditions indicated in S2 Table. Primers for *GSTs*, *GPx4a* and *GPx4b* genes are designed according to Fu & Xie [13] and Hermesz & Ferencz [12]. The reaction mixture (15μl) comprised of 7.5 μl of 2× SYBR^®^ Premix Ex Taq^™^ II (Takara), 2μl of diluted cDNA, forward and reverse primers, and RNase free dH_2_O. In order to assess target amplification specificity, melting curve analysis was performed over a range of 55–95°C. The threshold cycle (Ct) value was obtained from the CFX96^™^ Real-Time PCR system software (Bio-Rad). Each Ct value of target gene was normalized with corresponding Ct values of *β-actin*. The 2^-ΔΔCt^ method [44] was used to determine the relative expression levels of target genes.

### Western blotting

The processes for hepatopancreatic and intestinal protein extraction and western blotting were the same as those described by Hu et al. [45] and Jiang et al. [46]. Briefly, after extraction, the protein concentrations were determined with the Bio-Rad protein assay kit (Bio-Rad, Hercules, CA, USA). Equal amounts of protein were separated by sodium dodecyl sulfate polyacrylamide gel electrophoresis (SDS-PAGE) and transferred to a PVDF membrane. Membranes were blocked for 1 h at room temperature (RT) before being washed thrice with TBST (10 min each), and incubated with primary antibody overnight at 4°C. Anti-Nrf2 and β-actin antibodies were the same as those in previous studies from our laboratory [45,46]; these had been checked and also successfully cross-reacted with Jian carp proteins of interest. β-actin was used as control protein. Next, membranes were again washed three times before incubation with HRP-conjugated secondary antibody in TBST for 2 h. Immune complexes were visualized with an ECL kit (Millipore). Densitometric analyses of the protein bands were performed in Image J (NIH, USA). Different treatments were expressed relative to the level observed in the control group. The experiment was repeated at least three times, and similar results were obtained each time.

### Statistics

All data were subjected to one-way analysis of variance (ANOVA) followed by the Duncan’s method to determine significant differences among groups at the level of *P*<0.05 through SPSS 17.0 (SPSS Inc.).

## Results

### Contents of choline and PtdCho

As shown in Table 1, choline contents in hepatopancreas and intestine were significantly increased with dietary choline levels up to 607 mg/kg diet (*P*<0.05), however, they were decreased significantly once dietary choline level reached 1820 mg/kg diet (*P*<0.05). Similarly, PtdCho contents in hepatopancreas and intestine were enhanced significantly by increased dietary choline levels up to 607 and 310 mg/kg diet (*P*<0.05) respectively, and decreased significantly by choline of 1820 mg/kg diet (*P*<0.05).

**Table 1:** Effects of dietary choline (mg/kg diet) on choline and phosphatidylcholine (PtdCho) contents in the hepatopancreas and intestine of juvenile Jian carp^1^.

Choline	165	310	607	896	1167	1820
Choline (μg/g)						
Hepatopancreas	208.3±12.1^a^	241.6±16.9^ab^	282.6±14.3^bc^	295.9±23.9^c^	290.4±13.8^c^	238.0±8.6^ab^
Intestine	57.3±3.8^a^	82.4±5.0^b^	114.8±8.1^c^	116.9±6.2^c^	108.7±6.8^c^	89.3±4.5^b^
PtdCho (μg/g)						
Hepatopancreas	95.0±7.2^a^	111.5±12.9^a^	150.1±8.6^b^	151.3±4.4^b^	144.8±4.8^b^	105.6±7.5^a^
Intestine	1613.1±49.8^a^	1925.7±69.9^bc^	1986.6±127.5^c^	1996.9±70.6^c^	1871.4±40.9^bc^	1752.5±47.0^ab^

^1^ Values are means with S.E. of four replicates, with 5 fish in each replicate. Mean values with the different superscripts in the same row are significantly different (P<0.05).

### ROS contents, oxidative status and antioxidant enzyme activities

Hepatopancreatic ROS, MDA, PC and 8-OHdG decreased significantly with increasing dietary choline, resulting in the levels highest for fish fed the choline-deficient diet (*P*<0.05) (Table 2). The GSH content, CuZnSOD, MnSOD, CAT, GPx, GST and GR activities in hepatopancreas are also showed in Table 2. The GSH content in hepatopancreas was the highest in group fed with the choline-unsuppplemented diet and decreased with the increasing of dietary choline levels (*P*<0.05). The activities of CuZnSOD and MnSOD were significantly increased with the increase level of dietary choline and the highest in groups fed with 1820 and 1167 mg/kg diet respectively (*P*<0.05). The hepatopancreas GPx activity was significantly increased with the increment of dietary choline levels up to 310 mg/kg diet (*P*<0.05), and then a plateau. GST activity was significantly higher in the groups fed with 607, 896 and 1167 mg choline/kg diet than that in the choline-deficient group (*P*<0.05). However, dietary choline had no significant effects on CAT and GR activities in hepatopancreas (*P*>0.05). Regression analysis showed that CuZnSOD activity and GSH content in hepatopancreas quadratically responded to increasing dietary choline levels (Table 2).

**Table 2:** Effects of dietary choline (mg/kg diet) on oxidative damage and antioxidant parameters in the hepatopancreas of juvenile Jian carp^1^.

Choline	165	310	607	896	1167	1820
ROS	305.5±23.8^b^	199.9±9.8^a^	213.9±11.0^a^	213.9±24.8^a^	189.8±18.3^a^	190.0±23.3^a^
MDA	9.82±0.20^c^	8.39±0.20^a^	8.45±0.15^a^	8.45±0.27^a^	8.63±0.11^ab^	9.17±0.31^b^
PC	0.67±0.02^c^	0.56±0.01^a^	0.56±0.02^a^	0.60±0.02^ab^	0.63±0.03^bc^	0.66±0.02^bc^
8-OHdG	22.46±1.54^d^	3.37±0.29^a^	5.89±0.45^b^	6.85±0.30^b^	7.73±0.47^bc^	9.78±0.45^c^
CuZnSOD	6.68±0.20^a^	11.43±0.23^b^	17.78±0.40^c^	17.83±0.46^c^	18.84±0.47^cd^	19.89±0.44^d^
MnSOD	8.88±0.30^ab^	8.15±0.33^a^	10.92±0.32^c^	10.74±0.32^c^	12.67±0.40^d^	9.49±0.26^b^
CAT	30.41±1.26^a^	32.24±0.23^a^	31.55±0.76^a^	32.10±0.87^a^	31.55±0.83^a^	30.73±0.49^a^
GPx	85.14±2.75^a^	92.93±2.07^b^	93.09±2.36^b^	97.89±1.64^b^	97.38±3.12^b^	97.71±3.24^b^
GST	43.68±0.85^a^	45.61±1.74^ab^	50.41±0.69^c^	48.13±1.23^bc^	49.78±0.99^c^	43.82±1.11^a^
GR	38.45±1.22^a^	37.93±1.09^a^	38.13±1.10^a^	38.13±1.09^a^	37.93±1.09^a^	38.55±1.15^a^
GSH	9.54±0.29^d^	8.22±0.21^c^	7.53±0.24^b^	7.57±0.20^b^	6.18±0.26^a^	6.70±0.03^a^
Regression						
Y_CuZnSOD_ = -0.000008x^2^+0.02x+4.45					R^2^ = 0.923	*P* < 0.05
Y_GSH_ = 0.000002x^2^-0.005x+10.02					R^2^ = 0.884	*P* < 0.05

^1^ Values are means with S.E. of four replicates, with 5 fish in each replicate. Mean values with the different superscripts in the same row are significantly different (*P*<0.05).

ROS, reactive oxygen species (fluorescence intensity/mg protein); MDA, malondialdehyde (nmol/mg tissue); PC, protein carbonyl (nmol/mg protein); 8-OHdG, 8-hydroxydeoxyguanosine (ng/mg protein); CuZnSOD, Copper-zinc (U/mg protein); MnSOD, manganese superoxide dismutase (U/mg protein); CAT, catalase (U/mg protein); GPx, glutathione peroxidase (U/mg protein); GST, glutathione-S-transferase (U/mg protein); GR, glutathione reductase (U/g protein); GSH, glutathione (mg/g protein).

The effect of dietary choline on intestinal antioxidant parameters are displayed in Table 3. ROS, MDA, PC and 8-OHdG contents were the highest in the choline-deficient group (*P*<0.05). Intestinal CuZnSOD, MnSOD and CAT activities were significantly increased as the dietary choline levels enhanced and the highest in groups fed with 896, 607 and 896 mg/kg diet respectively (*P*<0.05) (Table 3). GPx activity was significantly improved by dietary choline levels of 1167 mg/kg diet (*P*<0.05), and GST activity was significantly higher in groups with choline levels of 310, 607 and 896 mg/kg diet (*P*<0.05), while GR activity showed no significant difference among dietary groups (*P*>0.05). However, GSH content was significantly decreased with the increase of dietary choline levels up to 310 mg/kg diet respectively (*P*<0.05), and then a plateau. Regression analysis again revealed a quadratic response of GPx to the incremental increase of dietary choline levels (Table 3).

**Table 3:** Effects of dietary choline (mg/kg diet) on oxidative damage and antioxidant parameters in the intestine of juvenile Jian carp^1^.

Choline	165	310	607	896	1167	1820
ROS	1611.7±77.2^b^	1432.6±65.4^ab^	1449.5±54.7^ab^	1426.9±60.0^ab^	1429.1±74.5^ab^	1301.4±82.1^a^
MDA	13.99±0.32^c^	11.31±0.45^a^	12.14±0.29^ab^	12.14±0.26^ab^	12.20±0.25^ab^	12.98±0.42^b^
PC	1.49±0.04^b^	1.33±0.05^a^	1.29±0.05^a^	1.25±0.05^a^	1.25±0.03^a^	1.26±0.04^a^
8-OHdG	98.90±8.08^c^	35.94±2.61^a^	32.85±3.04^a^	38.20±1.59^a^	39.40±1.30^a^	61.12±3.18^b^
CuZnSOD	4.77±0.13a	4.35±0.11a	7.21±0.23c	12.69±0.28e	8.47±0.17d	6.56±0.20b
MnSOD	3.47±0.09a	3.68±0.10a	8.78±0.29d	6.37±0.20c	4.64±0.15b	3.78±0.08a
CAT	1.96±0.05a	1.99±0.05a	2.53±0.07b	2.89±0.13c	2.46±0.08b	2.14±0.07a
GPx	60.22±1.56ab	62.39±1.40bc	62.87±1.85bc	64.41±1.68bc	65.68±2.13c	56.30±0.76a
GST	20.16±1.07a	25.79±0.86b	26.68±0.47b	26.68±0.92b	21.83±0.75a	20.14±0.42a
GR	48.62±1.45a	48.44±1.90a	52.97±1.39a	50.89±1.91a	52.33±1.32a	50.50±1.03a
GSH	14.93±0.53b	9.33±0.17a	8.46±0.11a	8.42±0.31a	8.95±0.32a	9.03±0.15a
Regression						
Y_GPx_ = -0.00001x^2^+0.02x+57.51					R^2^ = 0.914	*P* < 0.05

^1^ Values are means with S.E. of four replicates, with 5 fish in each replicate. Mean values with the different superscripts in the same row are significantly different (*P*<0.05).

ROS, reactive oxygen species (fluorescence intensity/mg protein); MDA, malondialdehyde (nmol/mg tissue); PC, protein carbonyl (nmol/mg protein); 8-OHdG, 8-hydroxydeoxyguanosine (ng/mg protein); CuZnSOD, Copper-zinc (U/mg protein); MnSOD, manganese superoxide dismutase (U/mg protein); CAT, catalase (U/mg protein); GPx, glutathione peroxidase (U/mg protein); GST, glutathione-S-transferase (U/mg protein); GR, glutathione reductase (U/g protein); GSH, glutathione (mg/g protein).

### Antioxidant enzyme gene expressions

**Fig 1:**
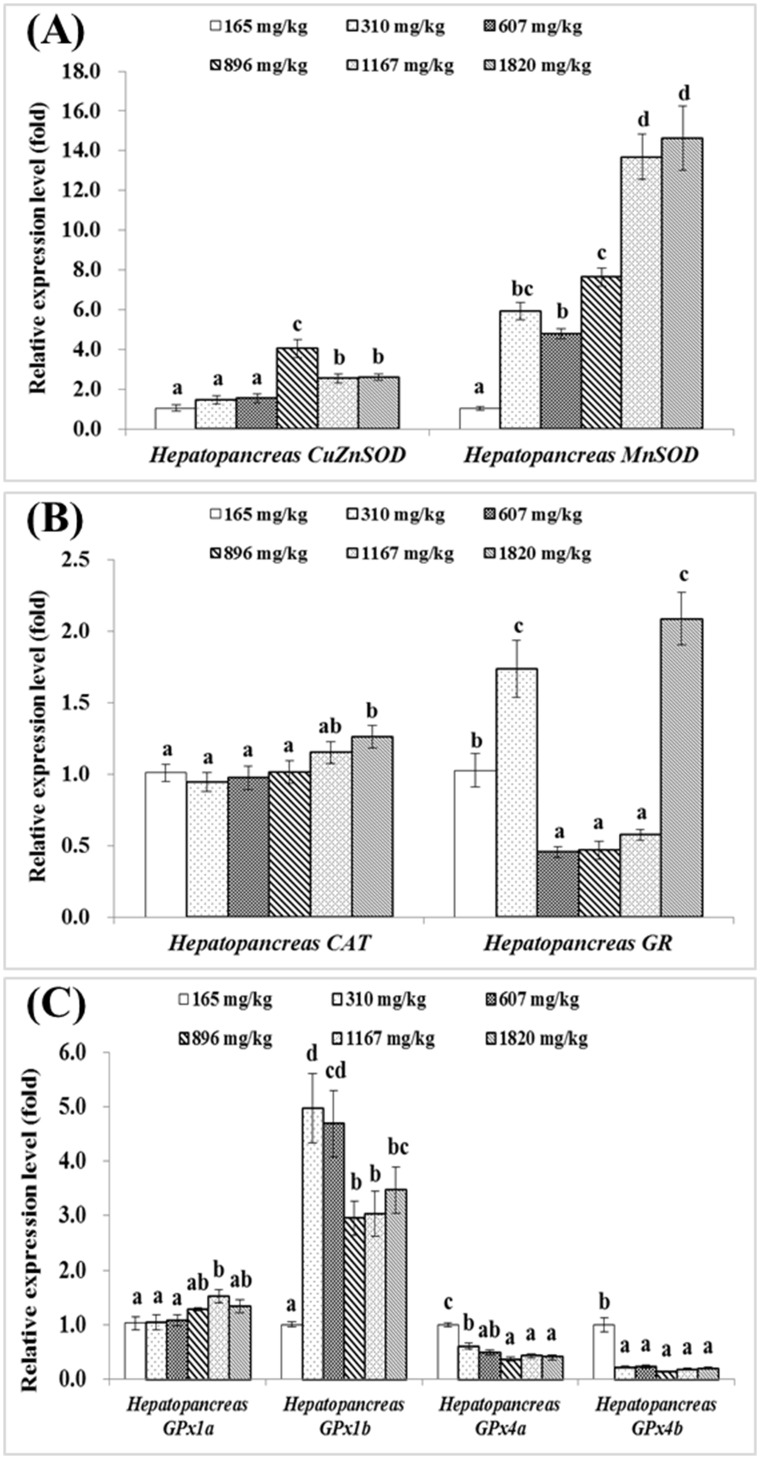
Effects of dietary choline (mg/kg diet) on *CuZnSOD*, *MnSOD*, *CAT*, *GR* and *GPx*s gene expressions in the hepatopancreas of juvenile Jian carp. A) *CuZnSOD* and *MnSOD*. B) *CAT* and *GR*. C) *GPx1a*, *GPx1b*, *GPx4a* and *GPx4b*. Values are means with S.E. of four replicates, with five fish in each replicate, and different letters denote significant difference (*P*<0.05).

**Fig 2:**
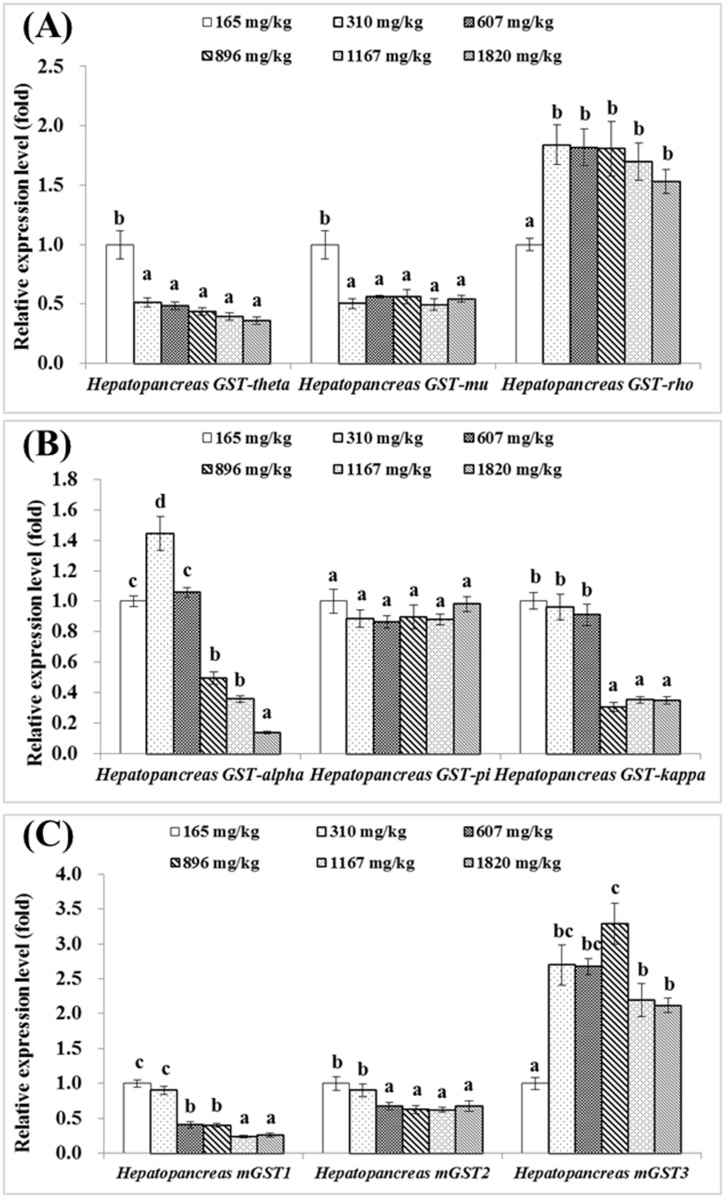
Effects of dietary choline (mg/kg diet) on *GSTs* gene expressions in the hepatopancreas of juvenile Jian carp. A) *GST-theta*, *GST-mu* and *GST-rho*. B) *GST-alpha*, *GST-pi* and *GST-kappa*. C) *mGST1*, *mGST2* and *mGST3*. Values are means with S.E. of four replicates, with five fish in each replicate, and different letters denote significant difference (*P*<0.05).

As presented in Fig 1A, expression levels of hepatopancreatic *CuZnSOD* and *MnSOD* were significantly upregulated by increased dietary choline level from 165 up to 896 and 1167 mg/kg diet respectively (*P*<0.05). Hepatopancreas *CAT* gene expression level showed no significant difference among groups fed with 165 to 1167 mg choline/kg diet (*P*>0.05), but was the highest in group fed with 1820 mg choline/kg diet (*P*<0.05) (Fig 1B). Hepatopancreas *GR* gene expression was the lowest in groups fed with 607, 896 and 1120 mg choline/kg diet (*P*<0.05) (Fig 1B). Gene expressions of hepatopancreas *GPx1a* and *GPx1b* were increased with increasing dietary choline level, and the highest in group fed with 1167 and 310 mg/kg diet respectively (*P*<0.05) (Fig 1C). However, the relative expression levels of *GPx4a* and *GPx4b* gene were significantly downregulated by choline, and the highest in the choline-deficient group (*P*<0.05) (Fig 1C). *GST-theta*, *GST-mu*, *mGST1* and *mGST2* gene expressions significantly downregulated with the increase of dietary choline levels up to 310, 310, 1167 and 607 mg/kg diet respectively (*P*<0.05), and plateaued thereafter (Fig 2). *GST-rho* gene expression was increased with the increased choline levels up to 310 mg/kg diet (*P*<0.05) and plateaued thereafter (Fig 2). The relative gene expression of *GST-alpha* was the highest in group fed with 310 mg choline/kg diet (*P*<0.05) (Fig 2). *GST-kappa* gene expression levels in groups fed with 896, 1167 and 1820 mg choline/kg diet were significantly lower than those in groups fed with the choline-deficient diet, 310 and 607 mg choline/kg diet (*P*<0.05) (Fig 2). *mGST3* gene expression significantly increased with the increased dietary choline levels up to 896 mg/kg diet, after then decreased significantly (*P*<0.05) (Fig 2). There is no significant difference in *GST-pi* gene expression among groups (*P*>0.05) (Fig 2). Regression analysis showed that *MnSOD*, *mGST1*, *mGST2*, *mGST3* and *CAT* gene expression levels were quadratic response to the increase of dietary choline levels (Table 4).

**Table 4:** Regression analysis of gene expressions responded to dietary choline.

Genes	Regressions	R^2^	*P*
Hepatopancreas			
*MnSOD*	Y = -0.000002x^2^+0.0125x-0.1104	0.8711	< 0.05
*mGST1*	Y = 0.0000005x^2^-0.0015x+1.2403	0.9515	< 0.05
*mGST2*	Y = 0.0000003x^2^-0.0009x+1.1219	0.9659	< 0.01
*mGST3*	Y = 0.0000004x^2^-0.0011x+1.189	0.9487	< 0.05
*CAT*	Y = 0.00000009x^2^-0.000003x+0.9664	0.8991	< 0.05
*Keap1a*	Y = 0.0000008x^2^-0.0021x+1.4323	0.904	< 0.05
Intestine			
*GPX1b*	Y = -0.000001x^2^+0.0028x+0.7496	0.9232	< 0.05
*GR*	Y = 0.0000007x^2^-0.0019x+1.3101	0.9372	< 0.05
*Keap1a*	Y = 0.0000008x^2^-0.002x+1.3131	0.880	< 0.05

**Fig 3:**
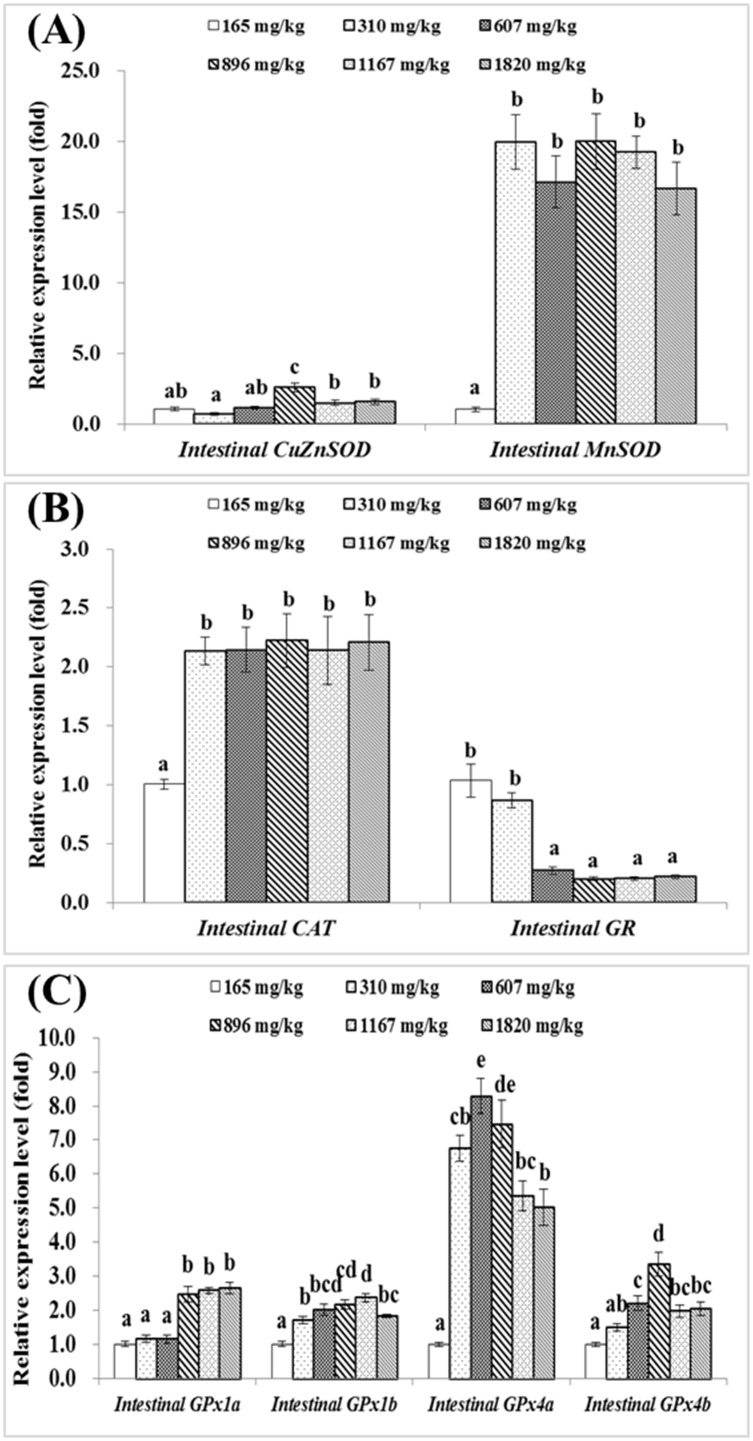
Effects of dietary choline (mg/kg diet) on *CuZnSOD*, *MnSOD*, *CAT*, *GR* and *GPx*s gene expressions in the intestine of juvenile Jian carp. A) *CuZnSOD* and *MnSOD*. B) *CAT* and *GR*. C) *GPx1a*, *GPx1b*, *GPx4a* and *GPx4b*.Values are means with S.E. of four replicates, with five fish in each replicate, and different letters denote significant difference (*P*<0.05).

**Fig 4:**
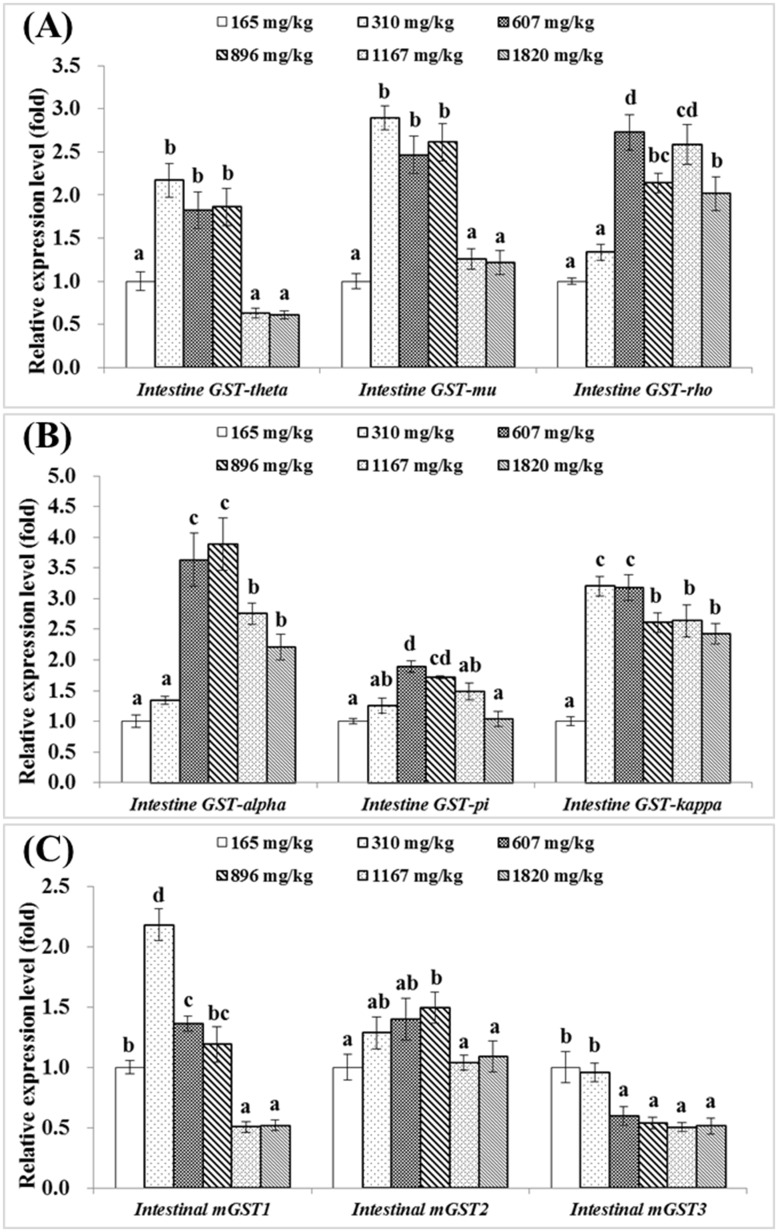
Effects of dietary choline (mg/kg diet) on *GSTs* gene expressions in the intestine of juvenile Jian carp. A) *GST-theta*, *GST-mu* and *GST-rho*. B) *GST-alpha*, *GST-pi* and *GST-kappa*. C) *mGST1*, *mGST2* and *mGST3*. Values are means with S.E. of four replicates, with five fish in each replicate, and different letters denote significant difference (*P*<0.05).

Intestinal *CuZnSOD* mRNA levels was the highest in the group with 896 mg choline/kg diet (*P*<0.05), while intestinal *MnSOD* mRNA levels were upregulated by dietary choline levels up to 310 mg/kg diet (*P*<0.05) before plateauing (Fig 3A). As showed in Fig 3B, intestinal *CAT* gene expression was the lowest in the choline-deficient group, and significantly upregulated by choline supplementation (*P*<0.05). However, the expression level of intestinal *GR* gene was significantly downregulated by dietary choline levels from 165 to 607 mg/kg diet (*P*<0.05), after then plateaued. Intestinal *GPx1a* gene expression level was significantly higher in groups fed with choline of 896, 1167 and 1820 mg/kg diet (*P*<0.05) (Fig 3C). Meanwhile, the relative expression levels of intestinal *GPx1b*, *GPx4a* and *GPx4b* gene also significantly increased with increasing dietary choline levels up to 1167, 607 and 896 mg/kg diet respectively, and then decreased significantly (*P*<0.05) (Fig 3C). The relative mRNA levels of *GST-pi*, *GST-rho*, *mGST1* and *mGST2* were significantly upregulated with the increased choline level up to 607, 607, 310 and 896 mg/kg diet respectively, and then downregulated significantly (*P*<0.05) (Fig 4). *GST-theta* and *GST-mu* gene expression levels in groups fed with 310, 607 and 896 mg choline/kg diet were significantly higher than those in other groups (*P*<0.05) (Fig 4). *GST-alpha* gene expression was the highest in groups fed with 607 and 896 mg choline/kg diet (*P*<0.05), and *GST-kappa* gene expression was the highest in groups fed with 310 and 607 mg choline/kg diet (*P*<0.05) (Fig 4). *mGST3* gene expression levels in groups fed with 165 and 310 mg choline/kg diet were significantly higher than those in other groups (*P*<0.05). Regression analysis suggested that the relative mRNA levels of *GPx1b* and GR were quadratic response to dietary choline levels (Table 4).

### Nrf2-Keap1 gene expressions

**Fig 5:**
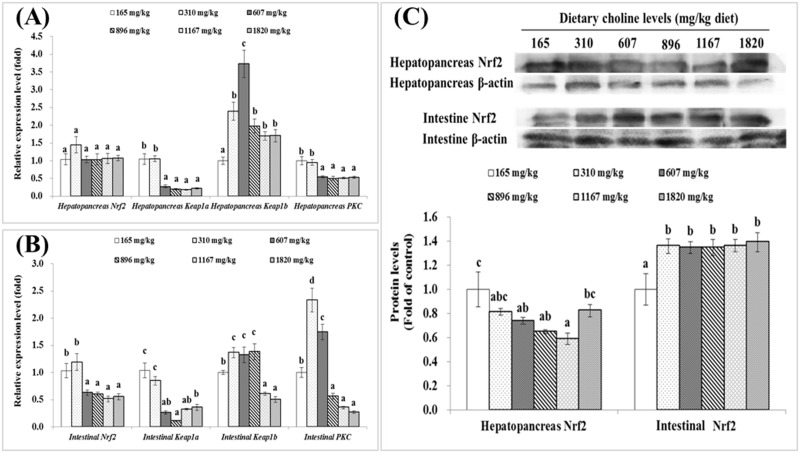
Effects of dietary choline (mg/kg diet) on *Nrf2*, *Keap1a*, *Keap1b* and PKC gene expressions, as well as Nrf2 protein levels in the hepatopancreas and intestine of juvenile Jian carp. A) Gene expressions in hepatopancreas. B) Gene expressions in intesine. C) Nrf2 protein levels. Values are means with S.E., and different letters denote significant difference (*P*<0.05).

Effects of dietary choline on the relative expressions of *Nrf2*, *Keap1a*, *Keap1b* and *PKC* gene in the hepatopancreas and intestine are showed in Fig 5. Expression level of *Nrf2* in hepatopancreas was not significantly different among groups (*P*>0.05). *Keap1a* and *PKC* mRNA levels in hepatopancreas were significantly downregulated by dietary choline levels up to 607 mg/kg diet and plateaued thereafter (*P*<0.05), while *Keap1b* gene expression was significantly upregulated by increased choline levels from 165 to 607 mg/kg diet (*P*<0.05), and downregulated significantly by further increase of choline levels up to 896 mg/kg diet (*P*<0.05). As shown in Fig 5B, mRNA levels of intestinal *Nrf2* was significantly decreased with dietary choline up to 607 mg/kg diet (*P*<0.05) and then plateaued. Intestinal *Keap1a* expression level was also downregulated by dietary choline and the lowest in group of 896 mg/kg diet (*P*<0.05). Intestinal *Keap1b* gene expression was the highest in groups fed with 310, 607 and 896 mg choline/kg diet (*P*<0.05). The relative expression levels of intestinal *PKC* gene was significantly increased with dietary choline levels up to 310 mg/kg diet and then decreased significantly (*P*<0.05). Expression levels of *Keap1a* in the hepatopancreas and intestine showed a quadratic response to dietary choline levels (Table 4). As presented in Fig 5C, Nrf2 protein levels in hepatopancreas were significantly decreased with dietary choline levels up to 1167 mg/kg diet (*P*<0.05), and then significantly increased (*P*<0.05). However, Nrf2 protein levels in intestine were significantly increased by dietary choline and the lowest in the choline-deficient group (*P*<0.05).

## Discussion

### Choline deficiency-induced oxidative damage and potential mechanisms of action in the hepatopancreas and intestine

In rat, choline deficiency decreased hepatic choline and choline metabolite concentrations [47]. Our present study showed that choline and PtdCho contents in the hepatopancreas and intestine were the lowest in the group fed with the basal diet, i.e. choline-deficient diet. Similarly, choline deficiency decreased choline concentration in the liver of cobia (*Rachycentron canadum*) [48] and throughout the body of hybrid tilapia (*Oreochromis niloticus* × *O*. *aureus*) [49]. In rat, choline deficiency also induces ROS generation in hepatocytes [5]. Excessive ROS can induce oxidative damage, resulting in lipid peroxidation, as well as protein and DNA oxidation [50]. Thus, we further investigated whether choline deficiency could induce oxidative damage in hepatopancreas and intestine and the potential underlying mechanism in fish.

#### Choline deficiency caused oxidative damage in the hepatopancreas and intestine

The present results showed that ROS, MDA, PC and 8-OHdG contents in the hepatopancreas and intestine were the highest in the choline-deficient group. The correlation analysis showed that 8-OHdG content was significantly positive related to ROS content in the hepatopancreas (r = +0.887, *P*<0.05), and PC content was significantly positive related to ROS content in the intestine (r = +0.851, *P*<0.05), suggesting that choline deficiency caused oxidative damage via increasing ROS levels in the hepatopancreas and intestine. As oxidative damage is usually associated with decreased antioxidant ability, we next investigated how choline deficiency influenced antioxidant capacity in the hepatopancreas and intestine.

#### Choline deficiency impaired hepatopancreatic and intestinal antioxidant systems

Glutathione is a major non-enzymatic antioxidant which can scavenge ROS directly or indirectly through enzymatic reactions [51]. Interestingly, the present study showed that GSH contents in the hepatopancreas and intestine were the highest in the choline-deficient group. This change might be attributed to two reasons. Firstly, because GSH content was increased by slight oxidative stress in fish [52], choline deficiency-induced oxidative stress may have contributed to this outcome. Secondly, the choline-deficient group may have consumed less GSH, a process that occurs via GPx and GST-catalyzed reactions [53]. In this study, the choline-deficient group exhibited decreased hepatopancreatic and intestinal GPx and GST activities than choline-supplemented groups (such as 607 and 896 mg/kg diet groups), suggesting lower GSH consumption in the choline-deficient group. However, further investigation is necessary to clarify the exact mechanisms behind these findings.

Besides, SOD and CAT are antioxidant enzymes that act as key lines of defense against ROS [54]. In this study, compared with choline-supplemented groups (such as 607 and 896 mg/kg diet groups), choline deficiency decreased hepatopancreatic and intestinal CuZnSOD and MnSOD activities, as well as intestinal CAT activity, indicating that choline deficiency-induced oxidative damage in both organs might be partially related suppressing antioxidant capacity in carp. Antioxidant enzyme activities in tissue were correlated with their mRNA levels in rat [55]. This study observed that choline deficiency caused decreases in the mRNA levels of intestinal *CAT*, hepatopancreatic and intestinal *CuZnSOD*, *MnSOD*, *GPx1a*, *GPx1b* and *GST-rho*, which might partly explain the decreased CAT, CuZnSOD, MnSOD, GPx and GST activities in the choline-deficient group. However, we also found that SOD isoforms were differentially susceptible to choline deficiency. These varied responses may be related to isoforms subcellular localization and choline function. CuZnSOD is found almost exclusively in intracellular cytoplasmic spaces, whereas MnSOD exists exclusively in the mitochondrial spaces of aerobic cells [54]. In rat, choline deficiency causes mitochondrial dysfunction in hepatocytes through disrupting mitochondrial transmembrane potential [56]. Thus, the importance of choline to mitochondrial may lead to the heightened sensitivity of *MnSOD* to choline deficiency; however, this hypothesis needs further investigation.

#### Choline deficiency altered Nrf2 expression in the hepatopancreas and intestine

The transcriptions of antioxidant enzyme genes are regulated by several cell signaling pathways [53]. The Nrf2-Keap1 signaling is found critical for regulating cellular antioxidant enzyme gene expressions in mammals [57] and zebrafish [58]. Thus, we further investigated the effects of choline on the Nrf2 signaling in the hepatpopancreas and intestine. The present study observed that choline deficiency upregulated mRNA levels of *Nrf2* but downregulated its protein levels in the intestine. However, choline deficiency upregulated Nrf2 protein levels in the hepatopancreas but showed no significant effect on its mRNA levels. Regoli & Giuliani [59] reported that the transcriptional induction of *Nrf2* gene could indicate an increase to its *de novo* synthesis in fish, which could explain our present results. Furthermore, choline deficiency downregulated *Keap1b* mRNA levels but upregulated *Keap1a* mRNA levels in the hepatpopancreas and intestine. These results might be partially related to the autoregulatory feedback loop in the Nrf2 pathway. In mammals, the Keap1 promoter contains a functional antioxidant response element sequence that allows Nrf2 regulation of *Keap1* expression [60]. Therefore, depressed expression of *Keap1b* gene might allow free-Nrf2 accumulation in the choline-deficient group, and the high *Nrf2* expression could then lead to increased *Keap1a* transcript level. However, the detailed mechanisms await further characterization.

### Choline deficiency induced varied change patterns of antioxidant system in different organs

#### Choline deficiency induced opposing changes to antioxidant enzymes in the digestive (hepatopancreas and intestine) versus immune (spleen) organ

Interestingly, the changes of antioxidant enzymes caused by choline deficiency in the present study were entirely opposite to our previous study in the carp spleen, which found that choline deficiency induced increase in activities of SOD, CAT, GPx and GST, and upregulation in the gene expressions of *CuZnSOD*, *MnSOD*, *CAT*, *GPx1a* and *GPx1a* [8]. These interesting results might be partially explained by the following reasons. First, in fish, the liver is the central metabolic organ, the intestine is the main site for nutrients digestion and absorption, and the spleen is one of the major immune organs [61]. It was reported that dampened antioxidant status decreased liver function [62] and intestinal absorption function in fish [63]. However, macrophage-derived ROS are critical for phagocytosis and subsequent destruction of microorganisms in terrestrial animal [64]. Additionally, choline is mainly absorbed in the intestine [27] and metabolized in the liver [26]. Thus, in the choline-supplemented groups, enhanced antioxidant ability in the hepatopancreas and intestine could benefit the maintenance of their normal function and continued choline metabolism, whereas decreased antioxidant enzymes activities in the spleen may benefit immune activation. Our previous study demonstrated that dietary choline improved immune function and attenuated inflammation in juvenile Jian carp [65], supporting this hypothesis. But the exact mechanisms underlying how the antioxidant ability in different organs is regulated by choline await further characterization.

#### Choline deficiency-induced change in antioxidant enzyme gene expressions differed between the hepatopancreas and intestine

Interestingly, although the activities of most antioxidant enzymes were generally decreased by choline deficiency, different change patterns of antioxidant enzyme gene isoforms were observed in the hepatopancreas and intestine of carp.

Firstly, the present study found that choline deficiency decreased mRNA levels of *GPx4a* and *GPx4b* in the intestine; whereas increased mRNA levels of *GPx4a* and *GPx4b* in the hepatopancreas. This interesting result might be partially explained by two factors. Tissue-specific expression of *GPx4* genes under oxidative stress is one likely reason for the observed differences. In carp, oxidative stress increased *GPx4a* transcript level in the liver, but reduced it in other tissue [12]. Another possible explanation is that increased mRNA levels of *GPx4a* and *GPx4b* in the hepatopancreas might be a mechanism to compensate for choline deficiency-induced membrane damage. In rat, choline deficiency firstly altered liver, while leaving other organs largely unaffected [28], and induced membrane peroxidation in the liver [66]. As GPx4 is the only GPx that can metabolize membrane phospholipid hydroperoxides [67], the liver might need more *de novo* synthesis of GPx4 for this reaction. But excessive ROS that resulted from choline deficiency may continually inactivate GPx activity, and we thus observed decrease in GPx activity.

Secondly, different expression patterns of varied *GST* isoforms caused by choline deficiency were observed in the hepatopancreas and intestine. In the present study, dietary choline deficiency decreased *mGST3* mRNA levels and increased *GST-theta*, *GST-mu*, *GST-kappa*, *mGST1* and *mGST2* mRNA levels in the hepatopancreas, but did the opposite in the intestine. To date, this is the first report about the regulation of *GSTs* gene expressions by choline in animal. In the hepatopancreas, increased mRNA levels of some *GST* isoforms might indicate an adaptive mechanism in the choline-deficient group; specifically, more *de novo* synthesis of those enzymes may be necessary for detoxifying oxidation products. The liver is the main organ for the metabolism of xenobiotics, and GSTs represent 10% of total cytosolic liver proteins in fish [68]. In river pufferfish, *GST-theta*, *GST-mu* and *GST-kappa* have the highest mRNA expressions in the liver and considered to be primarily associated with detoxification [14]. Mammalian mGST1, GST-kappa and GST-mu are also involved in protecting mitochondria from oxidative stress [69]. In rats, the liver is the most sensitive organ to choline deficiency [28], and mitochondria are susceptible to choline deficiency-induced oxidative damage [66]. Accordingly, the higher expressions of some hepatopancreatic *GSTs* genes may serve as a mechanism for protecting the hepatopancreas from dysfunctions related to choline deficiency, but this link requires further investigation.

#### Choline deficiency induced varied change patterns of antioxidant-related signaling molecule gene expressions between the hepatopancreas and intestine

The present study observed that the choline-deficient diet upregulated the intestinal *Nrf2* and *PKC* mRNA levels, but had no significant effect on the hepatopancreatic *Nrf2* mRNA levels and downregulated *PKC* mRNA levels in the hepatopancreas. The reason for this variability is unknown. But the highest *PKC* expression in the intestine may partly explain the highest intestinal *Nrf2* expression in the choline-deficient group.

### Optimal choline levels for antioxidant ability of juvenile jian carp

**Fig 6:**
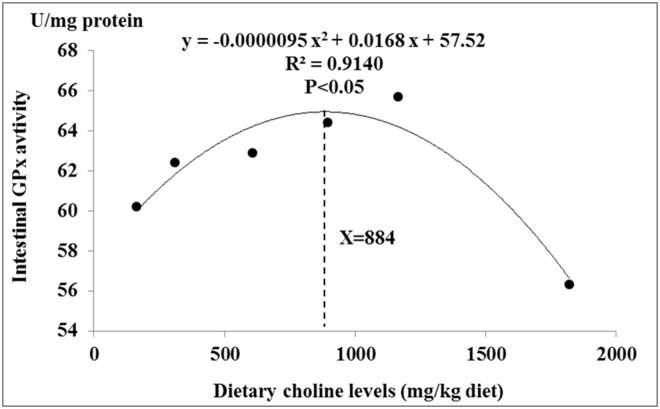
Quadratical regression analysis of intestinal GPx activity for juvenile Jian carp fed diets containing graded levels of choline for 65 days.

The current results showed that dietary choline deficiency could result in decrease of antioxidant ability of hepatopancreas and intestine in fish. Thus, it is quite necessary to evaluate the optimal choline levels required for antioxidant ability in the carp. Antioxidant-related parameters, such as antioxidant enzymes, have begun to be used to estimate the nutrient doses required for adequate function of the fish antioxidant system [70,71]. Based on intestinal GPx activity, the optimal choline level was 884 mg/kg diet for juvenile Jian carp (Fig 6), which was higher than the requirement (566 mg/kg diet) determined based on growth in our previous study [4].

## Conclusions

The present study showed that dietary choline deficiency induced oxidative damage in the hepatopancreas and intestine of Jian carp, mainly through decreasing enzymatic antioxidant capacity, i.e. CuZnSOD, MnSOD, GPx and GST activities which might be related to the downregulated mRNA levels of some antioxidant enzymes. Furthermore, the changes of antioxidant enzyme mRNA levels might be partly due to the alterations of Nrf2-Keap1 gene expressions induced by choline deficiency. However, this study provided three novel findings for understanding the effect of choline on antioxidant system in vertebrates. First, choline deficiency enhanced GSH contents probably by decreasing its consumption in the hepatopancreas and intestine. Second, this study firstly indicated that choline deficiency induced varied change patterns of different *GPx* and *GST* isoforms between the hepatopancreas and intestine. Third, this study firstly found that choline deficiency induced different antioxidant response in the hepatopancreas and intestine compared with the response in the spleen. Moreover, choline deficiency caused opposing changes in the mRNA levels of intestinal versus hepatopancreatic *GPx4*, *mGST3*, *GST-theta*, *GST-mu*, *GST-kappa*, *mGST1*, and *mGST2*. However, further investigation is necessary to fully understand the underlying mechanism of these choline-mediated antioxidant responses in the vertebrate liver and intestine.

## Supporting Information

S1 Table Composition and nutrients content of the basal diet. (DOC)

S2 Table Primer sequences of target genes and the housekeeping gene. (DOC)
