# Supplementary material for: A Comparative Study on Antioxidant System in Fish Hepatopancreas and Intestine Affected by Choline Deficiency: Different Change Patterns of Varied Antioxidant Enzyme Genes and Nrf2 Signaling Factors
Source: PLoS One. 2017 Jan 18;12(1):e0169888. doi: 10.1371/journal.pone.0169888 (PMC5242466; doi:10.1371/journal.pone.0169888)
Supplement: S1 Table — (DOC) [file pone.0169888.s001.doc]

**S1 Table**

**Composition and nutrients content of the basal diet.**

| Ingredients | g/kg diet | Nutrients content (g/kg diet)^1^ | |
| --- | --- | --- | --- |
| Fish meal | 20.0 | Crude protein | 315.3 |
| Soybean protein concentrate | 170.3 | Crude lipid | 44.0 |
| Casein | 180.3 | Crude ash | 48.1 |
| Rice protein meal | 33.5 | Available phosphorus | 6.6 |
| Gelatin | 38.2 | n-3 | 10.0 |
| DL-methionine (99%) | 4.2 | n-6 | 10.0 |
| Thr (98.5%) | 3.6 | Methionine | 11.3 |
| Fish oil | 26.8 | Cysteine | 2.00 |
| Soy bean oil | 16.7 |  |  |
| α-starch | 140.0 |  |  |
| Corn starch | 276.8 |  |  |
| Ca(H_2_PO_4_)_2_ | 19.1 |  |  |
| Choline-free vitamin premix^2^ | 10.0 |  |  |
| Trace mineral premix^3^ | 10.0 |  |  |
| Choline chloride premix^4^ | 30.0 |  |  |
| Ethoxyquin (30%) | 0.5 |  |  |
| cellulose | 20.0 |  |  |

^1^ Crude protein, crude fat, crude ash, methionine, cysteine and available phosphorus were measured value. n-3 and n-6 contents were calculated according to NRC [30] and Bell [31].

^2^ Per kilogram of choline-free vitamin premix(g/kg): retinyl acetate (500000 IU/g) 0.800g, cholecalciferol (500000 IU/g) 0.480g, DL-α-tocopherol acetate (500 g/kg) 20.000g, menadione (500 g/kg) 0.200g, cyanocobalamin (100 g/kg) 0.010g, D-biotin (200 g/kg) 0.500g, folic acid (960 g/kg) 0.521g, thiamin nitrate (980 g/kg) 0.104g, ascorhyl acetate (920 g/kg) 7.247g, niacin (980 g/kg) 2.857g, inositol (980 g/kg) 52.857g, calcium-D-pantothenate (980 g/kg) 2.511g, riboflavine (800 g/kg) 0.625g, pyridoxine hydrochloride (980 g/kg) 0.755g. All ingredients were diluted with corn starch to 1 kg.

^3^ Per kilogram of trace mineral premix (g/kg): CuSO_4_·5H_2_O (250.0 g/kg Cu) 1.201g, KI (38.0 g/kg I) 2.895g, MnSO_4_·H_2_O (318.0 g/kg Mn) 4.089g, NaSeO_3_ (10.0 g/kg Se) 2.500g, FeSO_4_·7H_2_O (197.0 g/kg Fe) 69.695g, ZnSO_4_·7H_2_O (225.0 g/kg Zn) 21.640g. All ingredients were diluted with CaCO_3_ to 1 kg.

^4^ Per kilogram of choline chloride premix (g/kg): each treatment group containing choline chloride 0g, 5.5170g, 17.2769g, 29.0368g, 40.7967g and 64.3166g, respectively. Each choline chloride mixture was diluted with corn starch to 1 kg.
