# Supplementary material for: A Comparative Study on Antioxidant System in Fish Hepatopancreas and Intestine Affected by Choline Deficiency: Different Change Patterns of Varied Antioxidant Enzyme Genes and Nrf2 Signaling Factors
Source: PLoS One. 2017 Jan 18;12(1):e0169888. doi: 10.1371/journal.pone.0169888 (PMC5242466; doi:10.1371/journal.pone.0169888)
Supplement: S2 Table — (DOC) [file pone.0169888.s002.doc]

**S2 Table**

Primer sequences of target genes and the housekeeping gene^1^.

| Genes | Forward (5′→3′) | Reverse (5′→3′) | Annealing temperature | Accession number |
| --- | --- | --- | --- | --- |
| *CuZnSOD* | TGGCGAAGAAGGCTGTTTGT | TTCACTGGAGACCCGTCACT | 60.4°C | JF342355 |
| *MnSOD* | CTGCCTGACCTTCCATACGA | CCTTAGCCAGTGCCTCTTGATA | 62°C | JF411603 |
| *CAT* | CTGGAAGTGGAATCCGTTTG | CGACCTCAGCGAAATAGTTG | 54°C | JF411604 |
| *GPx1a* | GTGACGACTCTGTGTCCTTG | AACCTTCTGCTGTATCTCTTGA | 60.4°C | JF411605 |
| *GPx1b* | TATGTCCGTCCTGGCAATGG | ATCGCTCGGGAATGGAAGTT | 60.4°C | JF411606 |
| *GPx4a* | CCTTCCCATCCCACCAGTTT | TGCGGAGTCACCGTTCACAT | 60°C | FJ656211 |
| *GPx4b* | TGTGCCCAAGCCAACGACT | TGCCGCAAGCTGAGTGTAG | 60°C | FJ656212 |
| *GST-alpha* | AGTTGAGCCGTGCTGACGTTCAC | CGGCTGGAGGAACTTGCTGA | 60°C | DQ411310 |
| *GST-theta* | TATGCTGTACCTGACTGAGAAGTT | TCCTCTACTGCATTGTCCATCTT | 60°C | DQ411315 |
| *GST-kappa* | GTCTCTCGGCCAGTGAGTTGGA | AAGAGCTCAGCCTTCCCGTTCA | 60°C | DQ411311 |
| *GST-mu* | GGAAACTGATGAAGCGCAGATGAG | ATCTTGTCCCCAGCAAACCACTT | 60°C | DQ411312 |
| *GST-pi* | GATCTGCCCAACCACCTCAAACC | CCGGGCAGCAATCTTATCCACAT | 60°C | DQ411313 |
| *GST-rho* | GTCATGGTCGCGCTGGAGGAGAA | AGATACAGACACGCGGCGAACGA | 60°C | DQ411314 |
| *mGST1* | GATGTGTTCCTGGCCTTCTCCACA | GAAGACCAATCACCACAAAGGGAA | 60°C | DQ411316 |
| *mGST2* | CGGGGCTGATTCCAAAACAGAAG | CGAGACTGCTGCGAGGAGAACTG | 60°C | DQ411317 |
| *mGST3* | GTATCCTATTGCCGCCTCTGTGTT | GAATGACTCCAAACAGCCCGATGT | 60°C | DQ411318 |
| *GR* | GAGAAGTACGACACCATCCA | CACACCTATTGAACTGAGATTGAG | 56°C | JF411607 |
| *Nrf2* | TTCCCGCTGGTTTACCTTAC | CGTTTCTTCTGCTTGTCTTT | 60°C | JX462955 |
| *Keap1a* | GCTCTTCGGAAACCCCT | GCCCCAAGCCCACTACA | 60°C | JX470752 |
| *Keap1b* | CTACAACCCCGAGAGAGACGA | GGAGGAGATGAAGCTCCAGAC | 60°C | JX470753 |
| *PKC* | AAATCCACCAAGCGACCT | CGAACCCTCCCACAGACG | 60°C | JX470751 |
| *β-actin* | CGTGATGGACTCTGGTGATG | TCGGCTGTGGTGGTGAAG | 60°C | M24113 |

^1^ *CuZnSOD*, copper-zinc superoxide dismutase; *MnSOD,* manganese superoxide dismutase; *CAT*, catalase; *GPx*, glutathione peroxidase; *GST*, glutathione-S-transferase; *GR*, glutathione reductase; *Nrf2*, nuclear factor erythoid 2-related factor 2; *Keap1*, Kelch-like ECH-associated protein 1; *PKC*, protein kinase C.
